# Excretion of urine extracellular vesicles bearing markers of activated immune cells and calcium/phosphorus physiology differ between calcium kidney stone formers and non-stone formers

**DOI:** 10.1186/s12882-021-02417-8

**Published:** 2021-06-01

**Authors:** Jiqing Zhang, Sanjay Kumar, Muthuvel Jayachandran, Loren P. Herrera Hernandez, Stanley Wang, Elena M. Wilson, John C. Lieske

**Affiliations:** 1 Department of Urology, Beijing Chaoyang Hospital, Capital Medical University, No.8 Gongti Nanlu,Chaoyang District, 100020 Beijing, China; 2 Department of Internal Medicine, Division of Nephrology and Hypertension, Mayo Clinic, 200 First Street SW, MN 55905 Rochester, USA; 3 Department of Life Science, School of Basic Science and Research, Sharda University, Knowledge Park III, 201310 UP Greater Noida, India; 4 Department of Internal Medicine, Division of Hematology Research, Mayo Clinic, 55905 Rochester, MN USA; 5 Department of Physiology & Biomedical Engineering, Mayo Clinic, 55905 Rochester, MN USA; 6 Department of Laboratory Medicine and Pathology, Mayo Clinic, 55905 Rochester, MN USA

**Keywords:** Calcium, Oxalate, Urinary extracellular vesicles, Inflammation, Phosphorus, Urinary stone disease

## Abstract

**Backgrounds:**

Previous studies have demonstrated that excretion of urinary extracellular vesicles (EVs) from different nephron segments differs between kidney stone formers and non-stone formers (NSFs), and could reflect pathogenic mechanisms of urinary stone disease. In this study we quantified selected populations of specific urinary EVs carrying protein markers of immune cells and calcium/phosphorus physiology in calcium oxalate stone formers (CSFs) compared to non-stone formers (NSFs).

**Methods:**

Biobanked urine samples from CSFs (*n* = 24) undergoing stone removal surgery and age- and sex- matched NSFs (*n* = 21) were studied. Urinary EVs carrying proteins related to renal calcium/phosphorus physiology (phosphorus transporters (PiT1 and PiT2), Klotho, and fibroblast growth factor 23 (FGF23); markers associated with EV generation (anoctamin-4 (ANO4) and Huntington interacting protein 1 (HIP1)), and markers shed from activated immune cells were quantified by standardized and published method of digital flow cytometry.

**Results:**

Urine excretion of calcium, oxalate, phosphorus, and calcium oxalate supersaturation (SS) were significantly higher in CSFs compared to NSFs (*P* < 0.05). Urinary excretion of EVs with markers of total leukocytes (CD45), neutrophils (CD15), macrophages (CD68), Klotho, FGF23, PiT1, PiT2, and ANO4 were each markedly lower in CSFs than NSFs (*P* < 0.05) whereas excretion of those with markers of monocytes (CD14), T-Lymphocytes (CD3), B-Lymphocytes (CD19), plasma cells (CD138 plus CD319 positive) were not different between the groups. Urinary excretion of EVs expressing PiT1 and PiT2 negatively (*P* < 0.05) correlated with urinary phosphorus excretion, whereas excretion of EVs expressing FGF23 negatively (*P* < 0.05) correlated with both urinary calcium and phosphorus excretion. Urinary EVs with markers of HIP1 and ANO4 correlated negatively (*P* < 0.05) with clinical stone events and basement membrane calcifications on papillary tip biopsies.

**Conclusions:**

Urinary excretion of EVs derived from specific types of activated immune cells and EVs with proteins related to calcium/phosphorus regulation differed between CSFs and NSFs. Further validation of these and other populations of urinary EVs in larger cohort could identify biomarkers that elucidate novel pathogenic mechanisms of calcium stone formation in specific subsets of patients.

**Supplementary Information:**

The online version contains supplementary material available at 10.1186/s12882-021-02417-8.

## Background

Urinary stone disease (USD) is common, painful, and costly to manage, affecting approximately 1 in 11 people in the United States and 5–15 % of the population worldwide[1–3]. Management of USD constitutes a significant portion of the patient load in urology clinics[4]. The majority (70–80 %) of urinary stones are composed of calcium oxalate (CaOx), often in combination with calcium phosphate[5].The 5 year recurrence rate after a first USD event can be as high as 50 %[6]. Many stones, especially idiopathic CaOx stones, appear to arise from subepithelial inner medullary calcium phosphate (CaP) crystal deposits called Randall’s plaques (RP)[2, 7, 8]. These interstitial apatite deposits appear to originate in basement membrane zones of the thin limb of Henle’s loop, and over time extend along the basement membrane of the thin limb to create apatite plaques beneath the papillary epithelium[7–10]. The plaques eventually breach the surface epithelium of renal papillae and extrude into the urinary space[2, 9, 10]. Once exposed, RP appear to serve as a nidus for deposition of protein and crystal layers from the urine in the renal pelvis, thus ultimately leading to anchored CaP and/or CaOx urinary stones[2, 8, 9].However, much is still not clear about the initiation and progression of RP and calcium stone formation. This lack of an in-depth mechanistic understanding has hindered the development of potential therapies [5].

Supersaturation within tubular fluid favors CaOx crystal formation. Once formed these crystals may adhere to and become internalized by tubular epithelial cells, and subsequently transmigrate into the interstitium[11]. These, crystals may activate interstitial mononuclear phagocytes including dendritic cells and macrophages that can release cytokines including interleukin-1β (IL-1β) to promote inflammatory cell transmigration and recruitment to the site of inflammation[11, 12]. Activation of nicotinamide adenine dinucleotide phosphate (NADPH), leucine-rich repeat (LRR), and NOD like pyrin domain-containing protein 3 (NLRP3) in macrophages can potentially aid in crystal dissolution, and *in vitro* studies suggest that CaOx crystals up to 200 μm in size can be dissolved within 3 days[7, 11]. NLRP3 serves as a marker of inflammasome activation, a process that promotes immune cell influx and triggers vascular permeability, leukocyte recruitment, complement activation, and inflammatory mediator production[13].

Extracellular vesicles (EVs) are lipid bilayer membrane-bound vesicles secreted by almost all cells involved in pathophysiological processes[12, 14]. EVs appear to transmit signals between cells under both physiological and pathological conditions[14]. The concentration and content of bioactive molecules in urinary EVs, including microRNA, DNA, mRNA, lipids and proteins all depend on their cell of origin and/or stimulus for their secretion [14–16]. Previous studies have reported that EVs participate in signal communication during renal regenerative and pathological processes[14]. Thus, specific populations of urinary EVs may reflect pathophysiological processes within the kidney[15]. In particular, urinary EVs released from the epithelium of different nephron segments could serve as biomarkers of diverse pathological states and response to therapeutic agents[5]. Previous reports demonstrated that EVs derived from specific immune cells and EVs carrying innate immune proteins can be detected in the urine after kidney transplantation[17, 18].Thus, in the present study we characterized populations of urinary EVs derived from activated immune cells in CSFs compared to NSFs (controls). Our previous studies demonstrated that urinary excretion of specific EV populations derived from different nephron segments varied between cohorts of CSFs and NSFs[5, 19].Specifically we quantified urinary EVs bearing markers of immune/inflammatory cells activation, calcium/phosphorus physiology and EV generation from the plasma membrane and endocytic vesicles.

## Methods

### Urine sample collection and storage

This study was approved by the Institutional Review Board at the Mayo Clinic, Rochester, MN. CSFs were recruited at the time of percutaneous nephrolithotomy (PCNL) for stone removal. Those CSFs with majority calcium oxalate stones and without secondary causes including hyperparathyroidism or enteric hyperoxaluria were included in the current study. All CSFs had preoperative stone protocol CT examinations available for review. Age- and sex- matched NSFs were recruited from the community lacked history of a clinical kidney stone event, but did not have imaging to exclude asymptomatic stones. Renal papillary surface area affected by RP was assessed via ureteroscopic video mapping at the time of percutaneous stone removal followed by quantitative image processing as described previously [9, 10, 20]. A papillary tip biopsy was obtained from a representative calyx at the time of mapping as previously described for 17 of the CSFs in the current study [21]. Hematoxylin and eosin and Yasue stained sections of the biopsies were semi quantitatively scored for the presence of intraluminal crystals, basement membrane crystals and interstitial inflammation by a renal pathologist (LPH) blinded to the clinical data. Urine samples (24-hr) were collected in toluene preservative from CSFs (n = 24, 15 males and 9 females) with low (< 5 % papillary surface area; *n* = 16, 8 males and 8 females) and high (≥ 5 % papillary surface area; n = 8, 7males and 1 female ) amounts of RP as determined at the time of urologic stone removal surgery, and from age-/sex-matched non-stone formers (NSFs; controls) in the general population (*n* = 21; 10 males and 11 females), as previously described [10, 21]. Urine from CSFs was collected a minimum of 6 weeks after the surgical procedure. Urine aliquots were centrifuged (2100* g* for 10 min) to remove urinary cells and larger protein aggregates prior to freezing at -80 °C for EV analysis. Urine biochemistries of the 24-hr urine samples were performed at the Mayo Clinic Renal Testing Laboratory using standard protocols as previously described [21].

### Chemicals, reagents, and antibodies

Recombinant annexin-V (microvesicle marker) and mouse anti-human cluster of differentiation 3 (CD3; marker of T-lymphocyte), CD14 (monocyte marker), CD15 (neutrophil marker), CD19 (B-lymphocyte marker), CD45 (total leukocytes marker), CD68 (macrophage marker), and CD138 (plasma cell marker) antibodies conjugated with fluorescein isothiocyanate (FITC) or R-phycoerythrin (PE) and TruCOUNT™ (4.2 μm) beads were purchased from BD Biosciences, San Jose, CA. Mouse anti-human CD319 (marker of plasma cell) antibody was purchased from BioLegend, San Diego, CA. FITC conjugated rabbit anti-human fibroblast growth factor 23 (FGF23) antibody was obtained from Biorbyt, Cambridge, Cambridgeshire, UK. PE conjugated rabbit anti-human Huntington interacting protein 1 (HIP1), anti-human SLC20A1 (phosphate transporter 1; PiT1), anti-human SLC20A2 (phosphate transporter 2; PiT2), and anti-human Klotho antibodies were from Lifespan Biosciences, Inc. Seattle, WA. FITC conjugated rabbit anti-human anoctamin-4 (ANO4) antibody was obtained from United States Biological, Salem, MA. HEPES (4-(2-hydroxyethyl)-1-piperazineethanesulfonic acid), and Hanks balanced salts were purchased from Sigma Chemicals, St. Louis, MO. All reagents and solvents used in this study were of analytical/reagent grade.

### Quantification of urinary EVs by flow cytometry

A standardized and validated flow cytometry (BD FACSCanto™) method was used to define EVs by size (≥ 200nm to ≤ 1000nm) and annexin-V-fluorescence for quantification of selected surface biomarker carrying urinary EVs as previously described in detail[5, 15, 16].The absolute number of urinary EVs positive for selected specific biomarkers is reported as both the number of EVs per µL of urine and also normalized to 24-hr urine creatinine concentration[5]. Normalization to urinary creatinine was used to account for the varied concentration of the timed urine collections[22].

### Statistical analysis

Continuous variables were expressed as the median, 25th and 75th percentile. The Wilcoxon rank sum test was used to identify significant differences between groups for continuous variables. Correlations between specific urinary EV populations and urinary phosphorus or calcium excretion was assessed using Spearman’s rank correlation coefficient. Nominal and categorical variables were compared using a chi-squared likelihood ratio or Fisher’s exact test. P < 0.05 was accepted as statistically significant. JMP Pro 13 statistical software (SAS Institute; Cary, NC) was used for all statistical analysis.

## Results

### Analysis of clinical characteristics

All stones removed from CSFs at PCNL were composed of a majority calcium oxalate. CSFs had a (median (25 %, 75 %)) of 2 (1, 5) clinical stone events and 4 (1,7) stones on preoperative imaging. There was no significant difference found in age, sex distribution, body mass index, and systolic/diastolic blood pressure between the CSFs and NSFs groups, or between the high and low RP-CSF patients among CSFs (Table 1). Urine pH in CSFs was markedly lower than NSFs (*P* < 0.05), whereas, as expected, urine calcium, oxalate, creatinine, phosphorus, and CaOx supersaturation (SS) were significantly higher in CSFs than NSFs (*P* < 0.05). By papillary tip mapping, 8 CSF had high (≥ 5 %) RP and 16 low (< 5 %) RP. Urine pH was significantly lower in high RP-CSFs than NSFs and low RP-CSFs(*P* < 0.05).Urine calcium was significantly higher in high RP-CSFs than NSFs (*P* < 0.05), but there was no difference between low RP-CSFs and NSFs (Table 1).

**Table 1:** Preoperative patient characteristics and 24-hours urine metabolic profile

Variables		NSF (*n* = 21)	High RP CSF (*n* = 8)	Low RP CSF (*n* = 16)	CSF (High + Low RP), (*n* = 24)
Age (years)		65.5 (57.4, 73.0)	69.2 (60.4, 72.2)	61.2 (50.1, 72.9)	63.9 (57.1, 72.2)
Sex n (%)	Male	10 (47.6 %)	7 (87.5 %)	8 (50 %)	15 (62.5 %)
	Female	11 (52.4 %)	1 (12.5 %)	8 (50 %)	9 (37.5 %)
Body mass index (kg/m^2^)		26.2 (24.5, 30.5)	30.5 (24.3, 34.9)	27.7 (24.4, 30.0)	28.7 (24.4, 32.4)
Systolic Blood Pressure (mmHg)		124 (116.0, 137.5)	130.5 (112.3, 133.5)	119 (114, 137.8)	126 (114, 136.8)
Diastolic Blood Pressure (mmHg)		72 (64.0, 82.0)	72 (62.0, 82.3)	69.5 (62.0, 77.0)	70 (62.0, 77.8)
Urine pH		6.4 (6.0, 7.0)	**5.5**^a^ **(5.2, 5.6)**	**6.2**^b^ **(5.7, 6.5)**	**6.0**^c^ **(5.4, 6.3)**
Urine calcium (mg/24hr)		158.3 (86.4, 192.3)	**255.5**^a^ **(132.5, 406.0)**	186.5 (103.2, 273.2)	**207.5**^c^ **(110.8, 330.0)**
Urine oxalate (mmol/24hr)		0.27 (0.22, 0.35)	**0.41**^**a,b**^ **(0.28, 0.64)**	0.28 (0.22, 0.36)	**0.30**^**c**^ **(0.25, 0.48)**
Urine chloride (mmol/24hr)		113.8 (73.4, 139.7)	**170.5**^a^ **(121.8, 233.3)**	**107**^b^ **(78.0, 153.8)**	117.8 (99.6, 189.3)
Urine citrate (mg/24hr)		625.3 (331.3, 756.8)	298.5 (206.3, 805.1)	557 (231.7, 690.5)	450.2 (218.8, 690.5)
Urine creatinine (mg/24hr)		869.2 (633.1, 1067.3)	**1790.5**^a^ **(1583, 2060)**	**1068**^b^ **(844.3, 1451.5)**	**1384.1**^c^ **(904.8, 1647.3)**
Urine osmolality (mOsm/kg)		458 (314.5, 731.5)	494 (126.3, 687)	363 (319.5, 472.8)	392.5 (319.5, 556.8)
Urine phosphorus (mg/24hr)		516.3 (371.5, 759.9)	**1204.5**^a^ **(1030, 1575.3)**	**747.8**^b^ **(483.1, 1008.3)**	**913.5**^c^ **(525.3, 1169.6)**
Urine potassium (mmol/24hr)		48.7 (36.7, 76.0)	80.5 (59.3, 86.8)	**45.4** **(33.1, 66.8**)^b^	59.5 (36.4, 78.8)
Urine sodium (mmol/24hr)		116.3 (72.1, 155.1)	**174**^a^ **(131.5, 257.5)**	**118.5**^b^ **(91, 191.8)**	144.5 (106.3, 215.7)
Urine sulfate (mmol/24hr)		15.4 (11.3, 27.5)	25 (12, 34)	11 (9, 24)	21.5 (10.3, 27.3)
Urine volume (ml, 24 h)		2087 (1597, 2529)	2388 (1596, 3222)	2095 (1454, 2788)	2115 (1454, 3064)
Urine calcium phosphate-brushite SS (DG)		−0.8 (− 1.7, 0.4)	-1.7 (-2.0, 0.1)	-0.1 (-1.7, 0.8)	-0.34 (-2.0, 0.6)
Urine calcium oxalate SS (DG)		1.1 (0.7, 1.6)	1.8 (1.2. 2.5)	1.6 (1.1, 2.3)	**1.6**^c^ **(1.1, 2.3)**

Data are presented as median (25th and 75th percentile)

*P* values in bold denote significance at < 0.05 level

Abbreviations: *CSF* calcium stone formers; *DG* delta Gibbs; *NSF* non-stone formers; *SS* supersaturation

^a^Significant difference between high RP CSF and NSFs

^b^Significant difference between high RP and low RP CSF

^c^Significant difference between CSFs and NSFs

### Total number of urinary EVs from activated immune/inflammatory cells

Urinary EVs derived from total leukocytes (CD45), neutrophils (CD15), and macrophages (CD68) were reduced in CSFs compared to NSFs (*P* < 0.05, Table 2 and Supplemental Figure 1), while excretions of EVs from activated T-lymphocytes (CD3),B-lymphocytes (CD19), monocytes (CD14)and plasma cells (CD138 plus CD319 positive) were not statistically different between CSFs and NSFs (Table 2).The number of total leukocyte (CD45) positive urinary EVs in high and low RP-CSF patients was lower than NSFs (*P* < 0.05). Urinary excretion of EVs bearing markers of immune cells did not statistically differ between high and low RP-CSFs (Table 2). In all cases, relative differences in the population of EVs were the same when expressed as EVs/µl (Supplemental Table 1).

**Table 2:** Urinary excretion of EVs carrying biomarkers of immune/ inflammatory cells in CSFs and NSFs

Urinary EVs/ mg creatinine	Marker	NSF (*n* = 21)	High RP CSF (*n* = 8)		Low RP CSF (*n* = 16)	CSF (High + Low RP) (*n* = 24)
Total leukocyte	CD45	10.5 (10.2, 11.9)	**10.1**^a^ **(9.4, 10.3)**	**10.1**^**b**^ **(9.5, 10.7)**		**10.1**^c^ **(9.5, 10.3)**
Neutrophil	CD15	11.7 (10.8, 12.6)	10.8 (10.0, 11.7)	10.8 (10.2, 11.5)		**10.8**^c^ **(10.2, 11.5)**
B-lymphocyte	CD19	11.0 (10.0, 12.3)	10.0 (9.7, 11.2)	10.0 (9.7, 11.2)		10.0 (9.7, 11.2)
T-lymphocyte	CD3	10.5 (10.1, 11.4)	10.3 (9.8, 10.4)	10.2 (9.7, 10.5)		10.2 (9.8, 10.5)
Monocyte	CD14	11.4 (10.1, 12.3)	10.4 (9.7, 11.2)	10.2 (9.5, 11.4)		10.3 (9.5, 11.2)
Macrophage	CD68	10.9 (10.4, 12.2)	**10.0**^a^ **(9.5, 10.7)**	10.4 (10.1, 10.9)		**10.3**^c^ **(9.8, 10.8)**
Plasma cell	CD138 + CD319	8.9 (7.8, 10.6)	8.8 (8.3, 9.4)	8.4 (7.5, 9.5)		8.7 (7.7, 9.5)

Data are presented as median (25th and 75th percentile) of natural log of EVs/mg creatinine. *P* values in bold denote significance at < 0.05 level

^a^Significant difference between high RP-CSF and NSFs

^b^Significant difference between low RP-CSF and NSFs

^**c**^Significant difference between CSFs and NSFs

### Urinary EVs expressing biomarkers of plasma membrane vesicle generation (anoctamin 4;ANO4) and endocytosis mediated exosome generation (Huntington interacting protein 1;HIP1)

Urinary excretion of ANO4 expressing EVs was significantly lower in CSFs than NSFs (*P* < 0.05), while EVs expressing HIP1 trended lower in CSFs compared to NSFs (*P* = 0.07, Table 3 and Supplemental Figure 2). There were no marked differences in the urinary excretion of EVs carrying HIP1 and ANO4 between low and high RP-CSF groups (Table 3). In all cases, relative differences in the population of EVs were the same when expressed as EVs/µl (Supplemental Table 2).

**Table 3:** Urinary excretion of EVs carrying biomarkers of calcium and phosphorus physiology in CSFs and NSFs

Urinary EVs/ mg creatinine	Marker	NSF (*n* = 21)	High RP CSF (*n* = 8)	Low RP CSF (*n* = 16)	CSF (High + Low RP) (*n* = 24)
Exosome generation	HIP1	11.7 (10.5, 12.7)	10.6 (10.2, 12.1)	10.8 (10.5, 11.4)	10.8 (10.3, 11.5)
Microvesicles generation	ANO4	12.0 (11.2, 13.0)	11.2 (10.2, 12.6)	11.3 (10.6, 12.3)	**11.3**^c^ **(10.5, 12.3)**
Calcium/phosphorus regulators	FGF23	11.5 (10.0, 12.0)	**9.8**^a^ **(9.4, 11.2)**	10.4 (9.8, 11.0)	**10.0**^c^ **(9.7, 11.0)**
Calcium/phosphorus regulators	Klotho	13.6 (13.0, 14.7)	12.9 (12.4, 14.3)	**12.5**^b^ **(11.6, 13.0)**	**12.5**^c^ **(11.7, 13.5)**
Phosphate transporter 1	PiT1	12.1 (10.8, 13.0)	**10.5**^a^ **(10.2, 11.7)**	**10.6**^b^ **(10.0, 11.6)**	**10.6**^**c**^ **(10.1, 11.6)**
Phosphate transporter 2	PiT2	12.8 (11.6, 14.7)	11.8 (11.1, 13.8)	11.6 (10.6, 13.0)	**11.7**^**c**^ **(10.9, 13.0)**

Data are presented as median (25th and 75th percentile) of natural log of EVs/mg creatinine. *P* values in bold denote significance at < 0.05 level

^a^Significant difference between high RP-CSF and NSFs

^b^Significant difference between low RP-CSF and NSFs

^**c**^Significant difference between CSFs and NSFs

### Urinary EVs expressing calcium/phosphorus physiology

Urinary excretions of EVs positive for FGF23, Klotho, PiT1, and PiT2 were significantly (*P* < 0.05) lower in CSFs compared to NSFs (Table 3 and Supplemental Figure 2).Urinary excretion of FGF23- carrying EVs in high RP-CSFs was significantly lower than NSFs (*P* < 0.05), while there was no significant difference between low RP-CSFs and NSFs (Table 3). In all cases, relative differences in the population of EVs were the same when expressed as EVs/µl (Supplemental Table 1). The number of PiT1 positive urinary EVs in high and low RP-CSF patients was lower than NSFs (*P* < 0.05). Urinary excretion of Klotho positive EVs were reduced in low RP-CSFs compared with NSFs (*P* < 0.05). There was no significant difference observed between high RP-CSF and low RP-CSF for PiT1, PiT2, FGF23 and Klotho (Table 3). Urinary excretion of EVs bearing PiT1 (*ρ*=-0.35;*P* < 0.05) and PiT2 (*ρ*=-0.36;*P* < 0.05) negatively correlated with 24-hr urine phosphorus excretion (Table 4), while excretion of EVs bearing FGF23 negatively correlated with 24-hr urine phosphorus (*ρ*=-0.34; *P* < 0.05, Table 4) and calcium (*ρ*=-0.29; *P* < 0.05, Table 5).

**Table 4:** Correlation between urine EVs carrying markers of EVs generation, calcium and phosphorous regulators and urine phosphorus

Urinary EV vs. Urine phosphorus correlation	Spearman’s Coefficient (ρ)	*P value*
Huntington interacting protein 1 (HIP1)	−0.19	0.20
Anoctamin 4 (ANO4)	−0.22	0.12
Fibroblast growth factor 23 (FGF23)	**−0.34**	**0.02**
Klotho	−0.21	0.15
Phosphate transporter 1 (PiT1)	**−0.35**	**0.02**
Phosphate transporter 2 (PiT2)	**−0.36**	**0.01**

**Table 5:** Correlation between urinary EVs carrying markers of EVs generation, calcium and phosphorous regulators and urine calcium

Urinary EV vs. urine Ca^++^ correlation	Spearman’s coefficient (ρ)	*P value*
Huntington interacting protein 1 (HIP1)	0.04	0.78
Anoctamin 4 (ANO4)	0.10	0.49
Fibroblast growth factor 23 (FGF23)	**-0.29**	**0.04**
Klotho	-0.08	0.59
Phosphate transporter 1 (PiT1)	-0.17	0.26
Phosphate transporter 2 (PiT2)	-0.08	0.60

In an exploratory analysis, the association of urinary EV populations with clinical stone events, stones on imaging, and papillary tip histology was examined (Table 6). Urinary EVs bearing HIP1 and ANO4 negatively (*P* < 0.05) correlated with clinical stones and basement membrane crystallization, while those bearing PiT1, PiT2, and Klotho negatively (*P* < 0.05) correlated with basement membrane crystallization. Urinary EVs bearing CD19 correlated positively with intraluminal crystals and interstitial inflammation.

**Table 6:** Correlation between specific populations of urinary extracellular vesicles (EVs), clinical stone events, and papillary tip histology in calcium kidney stone formers

Urinary EV population	Spearman’ correlation (ρ)	*P*-value
**Clinical Stones**		
Total Stone Events		
HIP1 positive EVs	-0.47	< 0.05
ANO4 positive EVs	-0.45	< 0.05
Stones on imagining at the time of surgery		
HIP1 positive EVs	-0.37	0.06
**Papillary Biopsy Findings**		
Intraluminal crystals		
CD19 positive EVs	0.59	< 0.05
Punctate basement membrane crystals		
HIP1 positive EVs	-0.44	< 0.05
ANO4 positive EVs	-0.57	< 0.05
Klotho positive EVs	-0.55	< 0.01
PiT1 positive EVs	-0.52	< 0.01
PiT2 positive EVs	-0.45	< 0.05
Dense basement membrane staining		
ANO4 positive EVs	-0.41	0.09
Interstitial inflammation		
CD19 positive EVs	0.56	< 0.05

Only significantly associated biomarker-positive urinary EVs are presented. Other biomarker-carrying urinary EVs did not correlate significantly with clinical stone events and papillary tip histology in this cohort of calcium kidney stone formers (data not shown)

## Discussion

In the current study, we quantified specific populations of EVs in the urine of CSFs compared to NSFs. Results indicate that the number of EVs carrying immune/inflammatory cell markers including those of leukocytes, neutrophils, and macrophages were lower in CSFs compared to NSFs. In addition, the number of EVs bearing markers of proteins important in calcium and phosphorus regulation including FGF23, PiT1, PiT2, and Klotho were also lower in the CSFs compared to NSFs. In general, the number of EVs did not differ between CSFs with high versus low amounts of RP. These results indicate that specific populations of urinary EVs may reflect ongoing pathological events in the kidney of the CSFs, but perhaps those pathways are independent of, or differ in some way, from pathways that resulted in RP formation.

Under normal conditions, nanocrystals can form and grow in tubular fluid, but then pass out as crystalluria[23]. Generally speaking, the literature suggests that on average stone formers excrete a greater number of crystals of larger size[23, 24]. Observations made using cultured cells *in vitro*, experimental animals *in vivo*, and kidney tissue from CSFs suggest that CaOx crystals can adhere to tubular epithelial cells, become transcytosed to the renal interstitium, and undergo dissolution within cells[8, 11, 12, 25–27]. The kidney harbors a variety of resident immune cells including macrophages and lymphocytes[28]. CaOx crystal deposition can activate renal immune cells to increase release of chemokines and pro-inflammatory cytokines, which in turn can recruit additional inflammatory cells including monocytes and neutrophils to the site[11, 12, 28, 29]. EVs secreted by these immune cells can serve as a biomarker of their presence and activation, and may also serve signaling functions *in vivo*, including antigen presentation, immune suppression, and tissue remodeling[30]. EVs secreted by innate immune cells such as macrophages appear to impact innate immune regulation primarily as pro-inflammatory and paracrine mediators[31]. In contrast, some subsets of immune cells and their signaling molecules can suppress an immune response[13, 32]. For example, neutrophils secrete EVs that have anti-inflammatory and immunosuppressive effects, mainly on dendritic cells and macrophages[31].

Although it is assumed that urinary EVs are mainly derived from kidney cells, evidence suggests that circulating exosomes can also enter the urine via trans tubular release[33]. Interestingly, in the current study the number of urinary EVs bearing immune cell markers CD45, CD15, and CD68 were reduced in CSFs compared with NSFs. Previously we had demonstrated that urinary excretion of EVs carrying the inflammatory mediator monocyte chemoattractant protein-1 was also lower in CSFs compared to NSFs[16]. Although in the current study sufficient quantities of matching kidney tissue was not available from the CSFs in order to quantitate sub populations of immune/inflammatory cell and correlate that with urinary EV populations, previously published studies do suggest that the number of immune cells within the kidney bearing CD68 may differ in CSFs compared to NSFs[27]. In the current study the number of inflammatory cell-derived EVs did not differ between high RP and low RP-CSFs. This finding is consistent with previous reports that RP is not associated with inflammation[8]. Thus, in this study the observed populations of EVs that differed between CSFs and NSFs likely reflect events involved in stone formation, but that are independent of RP formation and instead may relate to processing of those crystals that are retained in the kidney.

Singhto et al., [13, 29] reported that exposure of macrophages to calcium oxalate monohydrate (COM) crystals altered expression of 26 exosome proteins involved in immune signaling. They also demonstrated that exposure of macrophages with exosomes derived from COM-treated macrophages enhanced their COM binding capacity and increased crystal migration through the extracellular matrix[29].These macrophages manifest increased fragility due to actin cytoskeleton alterations[29]. To some extent, these findings may partially explain why in the current study the number of urinary EVs derived from immune cells was lower in CSFs than NSFs; however, further studies are needed to elucidate this mechanism.

In bone and cartilage the transmembrane proteins PiT1 and PiT2 transport inorganic phosphate (P_i_) into matrix vesicles, promoting nucleation and crystallization of Ca^2+^−PO_4_[34]. Fibroblast growth factor 23 (FGF23) is produced primarily by osteocytes[35]. FGF23 acts on the proximal tubule to decrease phosphorus reabsorption and reduce serum levels of 1,25-dihydroxyvitamin D3 [1,25(OH)2 Vitamin D3][35–37].The proximal tubule is responsible for reclaiming the majority of phosphorus filtered from the blood[38]. Klotho is a co-receptor that increases the binding affinity of FGF23 to its receptors[39]. In this study, urinary excretion of EVs carrying all four of these calcium and phosphorus related proteins (FGF23, Klotho, PiT1, and PiT2) were significantly lower in CSFs compared to NSFs. In addition, urinary excretion of FGF23- and PiT1-carrying EVs were significantly lower in high RP-CSFs compared to NSFs. Although the exact mechanisms are not clear, these results suggest that alterations and phosphorus transport in the proximal tubule may influence susceptibility to RP formation.

Urinary EVs are a mixture of exosomes and microvesicles[14]. Exosomes are formed within the endosomal network including early endosomes, late endosomes (multivesicular bodies, MVBs), and recycling endosomes[14]. Clathrin has been found in early endosomes, which form from clathrin-coated buds[40]. HIP1 recruits clathrin to endosomes through its central helical domain, which binds directly to highly conserved clathrin light chains (CLCs)[41]. HIP1 binding to CLC is necessary for HIP1 targeting to clathrin-coated pits and clathrin-coated vesicles[41]. Biogenesis of microvesicles occurs via outward budding and fission of the plasma membrane[14]. Anoctamin 4 (ANO4), a Ca^2+^-dependent phospholipid scramblase, not only takes part in exposing phosphatidylserine from the inner leaflet to the outer leaflet [42, 43], but also alters membrane curvature and facilitates EV release[43]. Thus, HIP1 and ANO4 play essential roles in membrane budding and EV formation and secretion[43]. PiT1 and PiT2 are present in matrix vesicles as noted above. We found that the numbers of EVs carrying plasma membrane EV-biogenesis markers (ANO4) were significantly lower in CSFs compared to NSFs, while the number of EVs carrying HIP1 also trended lower. It is possible that the reduced number of these matrix vesicles relates to their ongoing rupture and calcification within the interstitium of the CSFs.

Differences in the urinary biochemical profile of the CSF versus NSF group in the current study were not surprising, including lower urine pH and greater calcium and phosphorous excretion and calcium oxalate SS in CSF (Table 1) [4, 16, 27, 44]. Urine calcium and phosphorus excretion in high RP participants were markedly higher compared with NSFs, however those of low RP were not significantly different. Thus, metabolic factors may differ between high versus low RP-CSFs, as we have previously reported[21].We also found that EVs expressing PiT1 and PiT2 were negatively correlated with urinary phosphorus excretion, and urine excretion of EVs expressing FGF23 negatively correlated with urinary calcium and phosphate excretion. Thus, EVs containing FGF23, PiT1, and PiT2 may reflect underlying metabolic features that favor RP formation. However, further study will need to be completed to determine if quantification of EVs expressing PiT1, PiT2 and FGF23 will add additional clinically useful information to traditional urinary super saturation profiles, perhaps reflecting risk for RP and serving as a “liquid biopsy” for USD[5].

Our investigation has some limitations. The sample size is relatively small because we were limited to available biobanked urine samples from surgically mapped CSFs at Mayo Clinic, Rochester, MN. Thus, findings need to be verified in larger cohorts of CSFs. However, the results suggest that urinary EVs differ between CSFs and NSFs may influence directly or indirectly USD pathogenesis.

## Conclusions

In this study we demonstrated that the urinary excretion of EVs carrying immune cell markers including CD45, CD15, and CD68 were lower in CSFs compared to NSFs, but did not differ in the CSFs by RP amount. Excretion of EVs bearing proteins related to renal calcium/phosphorus physiology (FGF23, Klotho, PiT1, and PiT2) were also reduced in CSFs. These differences may reflect ongoing pathogenic events in both low and high RP-CSFs. The cause of the reduced number of these EVs associated with specific biomarkers related to USD risk, and their association with stone pathogenic mechanism(s), needs further study in larger cohorts of CSFs.

## Supplementary Information

**Additional file 1:**

**Additional file 2:**

**Additional file 3:**

## Data Availability

The datasets used and/or analyzed during the current study are available from the corresponding author on reasonable request.

## Abbreviations

ANO4: Anoctamin-4
CaOx: Calcium oxalate
CaOx SS: Calcium oxalate Supersaturation
CD: Cluster of differentiation
CLCs: Clathrin light chains
COM: Calcium oxalate monohydrate
CSFs: Calcium stone formers
DG: Delta Gibbs free energy unit
EVs: Extracellular vesicles
FGF23: Fibroblast growth factor 23
FITC: Fluorescein isothiocyanate
HIP1: Huntington interacting protein 1
LRR: Leucine-rich repeat
MCP-1: Monocyte chemoattractant protein-1
NADPH: Nicotinamide adenine dinucleotide phosphate
NLRP3: NOD like family pyrin domain-containing protein 3
NSFs: Non-stone formers
PE: Phycoerythrin
PiT1: Phosphate transporter 1
PiT2: Phosphate transporter 2
