# Supplementary material for: Excretion of urine extracellular vesicles bearing markers of activated immune cells and calcium/phosphorus physiology differ between calcium kidney stone formers and non-stone formers
Source: BMC Nephrol. 2021 Jun 1;22:204. doi: 10.1186/s12882-021-02417-8 (PMC8170929; doi:10.1186/s12882-021-02417-8)
Supplement: Supplementary file 1 — Additional file 1: [file 12882_2021_2417_MOESM1_ESM.docx]

**Table 1.** Urinary excretion of EVs carrying biomarkers of different immune/ inflammatory cells and calcium and phosphorus regulators from CSFs and NSFs.

| Urinary EVs/ µL urine | Marker | NSF  (n=21) | High RP CSF  (n=8) | | Low RP CSF (n=16) | CSF  (High+Low RP) (n=24) |
| --- | --- | --- | --- | --- | --- | --- |
| Total leukocyte | CD45 | 3.0  (2.2, 3.8) | **2.9**^c^  **(2.7, 3.2)** | **2.6^b^**  **(2.2, 2.8)** | | **2.7**^c^  **(2.3, 3.1)** |
| Neutrophil | CD15 | 4.0  (3.2, 4.7) | **3.8** ^c^  **(3.2, 4.5)** | 3.5  (2.7, 4.4) | | **3.1**^c^  **(2.4, 3.4)** |
| B-lymphocyte | CD19 | 3.2  (2.1, 4.1) | 3.2  (2.6, 3.8) | 3.1  (2.3, 3.2) | | 3.2  (2.6, 3.8) |
| T-lymphocyte | CD3 | 2.9  (2.3, 3.4) | 3.0  (2.2, 3.6) | 2.6  (2.0, 3.4) | | 2.8  (2.1, 3.5) |
| Monocyte | CD14 | 3.6  (2.4, 4.3) | 3.3  (2.5, 4.3) | 3.1  (2.3, 3.7) | | 3.2  (2.4, 3.8) |
| Macrophage | CD68 | 3.4  (2.5, 4.2) | **2.9** ^c^  **(2.4, 3.5)** | 3.2  (2.4, 3.7) | | **3.1** ^c^  **(2.4, 3.5)** |
| Plasma cell | CD138+CD319 | 1.4  (0.1, 2.4) | 1.8  (1.5, 2.1) | 1.2  (-.0.2, 2.0) | | 1.4  (-0.1, 2.1) |

Data are presented as median (25^th^ and 75^th^ percentile) of natural log of EVs/µl urine.

P values in bold denote significance at < 0.05 level.

^a^Significant difference between high RP-CSF and NSFs.

^b^Significant difference between low RP-CSF and NSFs.

**^c^**Significant difference between CSFs and NSFs.
