# Supplementary material for: Excretion of urine extracellular vesicles bearing markers of activated immune cells and calcium/phosphorus physiology differ between calcium kidney stone formers and non-stone formers
Source: BMC Nephrol. 2021 Jun 1;22:204. doi: 10.1186/s12882-021-02417-8 (PMC8170929; doi:10.1186/s12882-021-02417-8)
Supplement: Supplementary file 2 — Additional file 2: [file 12882_2021_2417_MOESM2_ESM.docx]

**Table 2.**Urinary excretion of EVs carrying biomarkers of calcium and phosphorus physiology from CSFs and NSFs.

| Urinary EVs/ µL urine | Marker | NSF  (n=21) | High RP CSF (n=8) | Low RP CSF (n=16) | | CSF  (High+Low RP) (n=25) | | |
| --- | --- | --- | --- | --- | --- | --- | --- | --- |
| Exosome generation | HIP1 | 3.8  (2.9, 4.3) | 3.8  (3.2, 4.7) | | 3.4  (2.8, 3.7) | | 3.6  (2.3, 3.8) |  |
| Microvesicles generation | ANO4 | 4.4  (3.6, 5.2) | **4.1**^c^  **(3.4, 5.3)** | | 3.9  (2.9, 4.8) | | **4.0**^c^  **(3.2, 5.0)** |  |
| Calcium/phosphorus regulators | FGF23 | 3.5  (2.5, 4.4) | **3.0**^c^  **(2.6, 3.7)** | | 2.8  (2.1, 3.6) | | **2.9**^c^  **(2.3, 3.5)** |  |
| Calcium/phosphorus regulators | Klotho | 6.0  (5.2, 6.8) | **5.9** ^c^  **(4.5, 6.8)** | | **4.9**^b^  **(3.9, 5.7)** | | **5.3**^c^  **(4.1, 6.2)** |  |
| Phosphate transporter 1 | PiT1 | 4.1  (3.0, 5.4) | **3.7 ^c^**  **(3.1, 4.5)** | | **3.5**^b^  **(2.6, 4.0)** | | **3.5 ^c^**  **(2.7, 4.2)** |  |
| Phosphate transporter 2 | PiT2 | 5.3  (4.2, 6.3) | **4.9 ^c^**  **(3.1, 4.7)** | | 4.4  (3.1, 6.0) | | **4.6^c^**  **(3.5, 5.9)** |  |

Data are presented as median (25^th^ and 75^th^ percentile) of natural log of EVs/µl urine.

P values in bold denote significance at <0.05 level.

^a^Significant difference between high RP-CSF and NSFs.

^b^Significant difference between low RP-CSF and NSFs.

**^c^**Significant difference between CSFs and NSFs.
