# Supplementary material for: Excretion of urine extracellular vesicles bearing markers of activated immune cells and calcium/phosphorus physiology differ between calcium kidney stone formers and non-stone formers
Source: BMC Nephrol. 2021 Jun 1;22:204. doi: 10.1186/s12882-021-02417-8 (PMC8170929; doi:10.1186/s12882-021-02417-8)
Supplement: Supplementary file 3 — Additional file 3: [file 12882_2021_2417_MOESM3_ESM.pptx]

## Slide 1
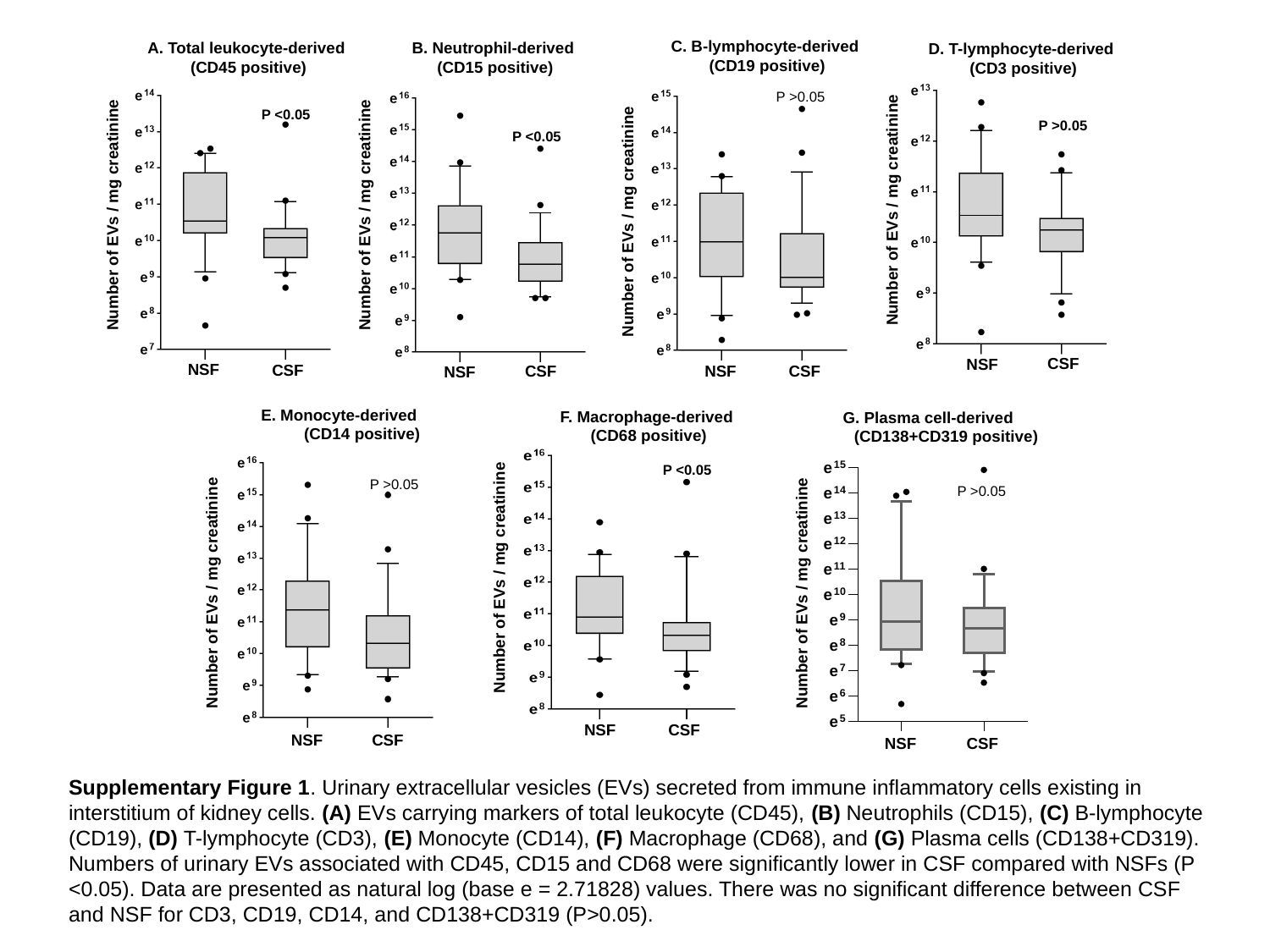

C. B-lymphocyte-derived
 (CD19 positive)
CSF
NSF
P >0.05
 Number of EVs / mg creatinine
A. Total leukocyte-derived
 (CD45 positive)
NSF
CSF
P <0.05
 Number of EVs / mg creatinine
B. Neutrophil-derived
 (CD15 positive)
CSF
NSF
P <0.05
 Number of EVs / mg creatinine
D. T-lymphocyte-derived
 (CD3 positive)
CSF
NSF
P >0.05
 Number of EVs / mg creatinine
E. Monocyte-derived
 (CD14 positive)
NSF
CSF
P >0.05
 Number of EVs / mg creatinine
F. Macrophage-derived
 (CD68 positive)
P <0.05
CSF
NSF
 Number of EVs / mg creatinine
G. Plasma cell-derived
 (CD138+CD319 positive)
P >0.05
NSF
CSF
 Number of EVs / mg creatinine
Supplementary Figure 1. Urinary extracellular vesicles (EVs) secreted from immune inflammatory cells existing in interstitium of kidney cells. (A) EVs carrying markers of total leukocyte (CD45), (B) Neutrophils (CD15), (C) B-lymphocyte (CD19), (D) T-lymphocyte (CD3), (E) Monocyte (CD14), (F) Macrophage (CD68), and (G) Plasma cells (CD138+CD319). Numbers of urinary EVs associated with CD45, CD15 and CD68 were significantly lower in CSF compared with NSFs (P <0.05). Data are presented as natural log (base e = 2.71828) values. There was no significant difference between CSF and NSF for CD3, CD19, CD14, and CD138+CD319 (P>0.05).

## Slide 2
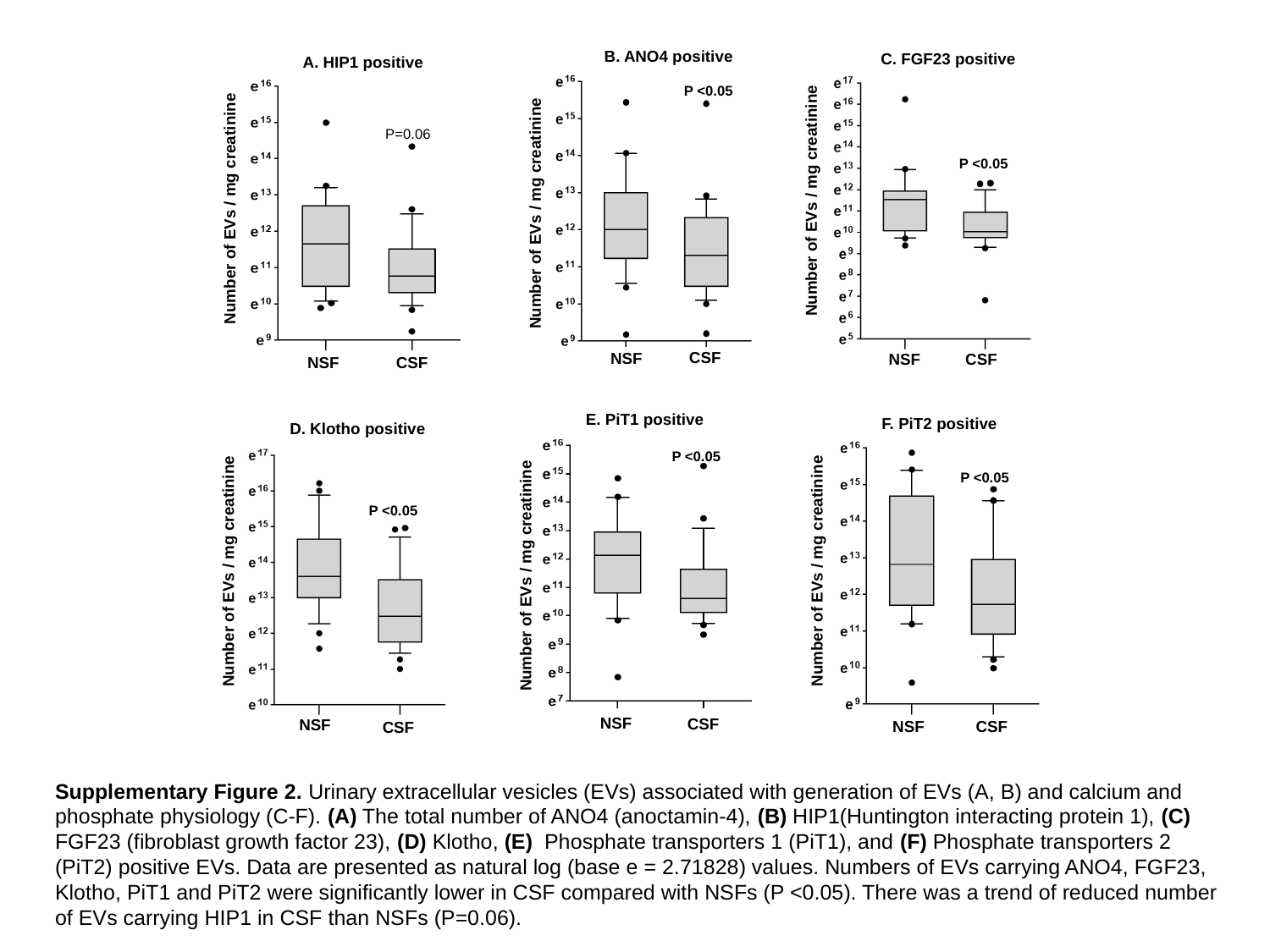

B. ANO4 positive
P <0.05
 Number of EVs / mg creatinine
CSF
NSF
C. FGF23 positive
P <0.05
 Number of EVs / mg creatinine
NSF
CSF
A. HIP1 positive
P=0.06
 Number of EVs / mg creatinine
NSF
CSF
E. PiT1 positive
P <0.05
 Number of EVs / mg creatinine
NSF
CSF
F. PiT2 positive
P <0.05
 Number of EVs / mg creatinine
NSF
CSF
D. Klotho positive
P <0.05
 Number of EVs / mg creatinine
NSF
CSF
Supplementary Figure 2. Urinary extracellular vesicles (EVs) associated with generation of EVs (A, B) and calcium and phosphate physiology (C-F). (A) The total number of ANO4 (anoctamin-4), (B) HIP1(Huntington interacting protein 1), (C) FGF23 (fibroblast growth factor 23), (D) Klotho, (E) Phosphate transporters 1 (PiT1), and (F) Phosphate transporters 2 (PiT2) positive EVs. Data are presented as natural log (base e = 2.71828) values. Numbers of EVs carrying ANO4, FGF23, Klotho, PiT1 and PiT2 were significantly lower in CSF compared with NSFs (P <0.05). There was a trend of reduced number of EVs carrying HIP1 in CSF than NSFs (P=0.06).
